# Targeting NF-κB Signaling in Preterm Labor: Molecular Mechanisms and Therapeutic Perspectives

**DOI:** 10.3390/ijms27177673

**Published:** 2026-08-27

**Authors:** Mikołaj Marynowski, Dominika Mech, Alicja Mastej, Angelika Masiarz, Żaneta Kimber-Trojnar

**Affiliations:** 1 Student’s Scientific Association at the Chair and Department of Obstetrics and Perinatology, Medical University of Lublin, 20-090 Lublin, Poland; dominika-mech@wp.pl (D.M.); ala.mastej72@gmail.com (A.M.); masiarz.ang@gmail.com (A.M.); 2 Chair and Department of Obstetrics and Perinatology, Medical University of Lublin, 20-090 Lublin, Poland

**Keywords:** preterm birth, nuclear factor kappa B, inflammation, myometrium, tocolysis, nuclear factor kappa B inhibitors

## Abstract

Preterm labor is a leading cause of neonatal morbidity and mortality, with inflammation playing a central role in its pathogenesis. This structured literature review summarizes the role of the nuclear factor kappa B (NF-κB) signaling pathway in term and preterm labor and critically evaluates current evidence on natural and synthetic NF-κB modulators as potential therapeutic approaches. The narrative overview covers publications through June 2026. Experimental, translational, and review studies were synthesized narratively. NF-κB integrates hormonal, mechanical, infectious, and sterile inflammatory signals in gestational tissues, promoting the expression of pro-inflammatory cytokines, cyclooxygenase-2, prostaglandins, chemokines, and contraction-associated proteins that drive uterine activation. While tightly regulated NF-κB activation is essential for physiological term labor, it’s premature or excessive activation contributes to inflammation-associated preterm labor. Natural compounds and synthetic agents consistently attenuate excessive NF-κB signaling and downstream inflammatory responses in preclinical models, supporting their potential as candidate therapeutic strategies. However, clinical evidence remains limited. Future research should prioritize tissue-specific, context-dependent modulation of NF-κB and establish the safety, optimal therapeutic windows, and clinical efficacy of NF-κB-targeted interventions during pregnancy.

## 1. Introduction

Preterm birth (PTB) is a prevalent condition and represents a major global public health concern. The incidence of PTB varies between countries, ranging from 4% to 16% of all live births. In 2020, approximately 13.4 million infants were born preterm, accounting for nearly 11% of all live births. PTB contributes to approximately 18% of deaths in children under five years of age and up to 35% of all neonatal deaths. Beyond increased mortality, PTB remains a leading cause of long-term morbidity, including hearing loss, blindness, chronic respiratory disorders, and central nervous system injuries [1,2,3]. Recent evidence indicates that the initiation of labor—both at term and preterm—is closely associated with the activation of inflammatory pathways. The transcription nuclear factor kappa B (NF-κB) acts as a central molecular integrator of immune, mechanical, and inflammatory signals in gestational tissues. NF-κB signaling is not inherently pathological in pregnancy: basal and tightly regulated NF-κB activity is required for placental development, maternal–fetal immune tolerance, and the initiation of normal parturition. However, excessive, dysregulated, or prematurely activated NF-κB signaling can trigger pathological inflammatory cascades and induce myometrial contractility independently of the physiological timing of labor. Despite the availability of tocolytic therapies, which primarily target downstream uterine contractions, their effectiveness in preventing PTB remains limited. This underscores the need for novel therapeutic strategies targeting upstream inflammatory mechanisms driving preterm labor [1,2,3]. The aim of this study is to summarize the role of the NF-κB pathway in the regulation of term and preterm labor and to discuss potential therapeutic strategies for selective modulation of NF-κB signaling. The goal of such modulation would be to inhibit premature myometrial activation while preserving the essential physiological functions of this pathway during pregnancy.

## 2. Immunological Basis of Labor

### 2.1. Labor as an Inflammatory Process

Labor, both at term and preterm, is a physiological process closely associated with activation of inflammatory pathways. During normal pregnancy, the myometrium (myometrial smooth muscle cells, mSMCs) remains in a state of functional quiescence for most of gestation due to dominant anti-inflammatory signaling and the action of steroid hormones, primarily progesterone.

**Figure 1:**
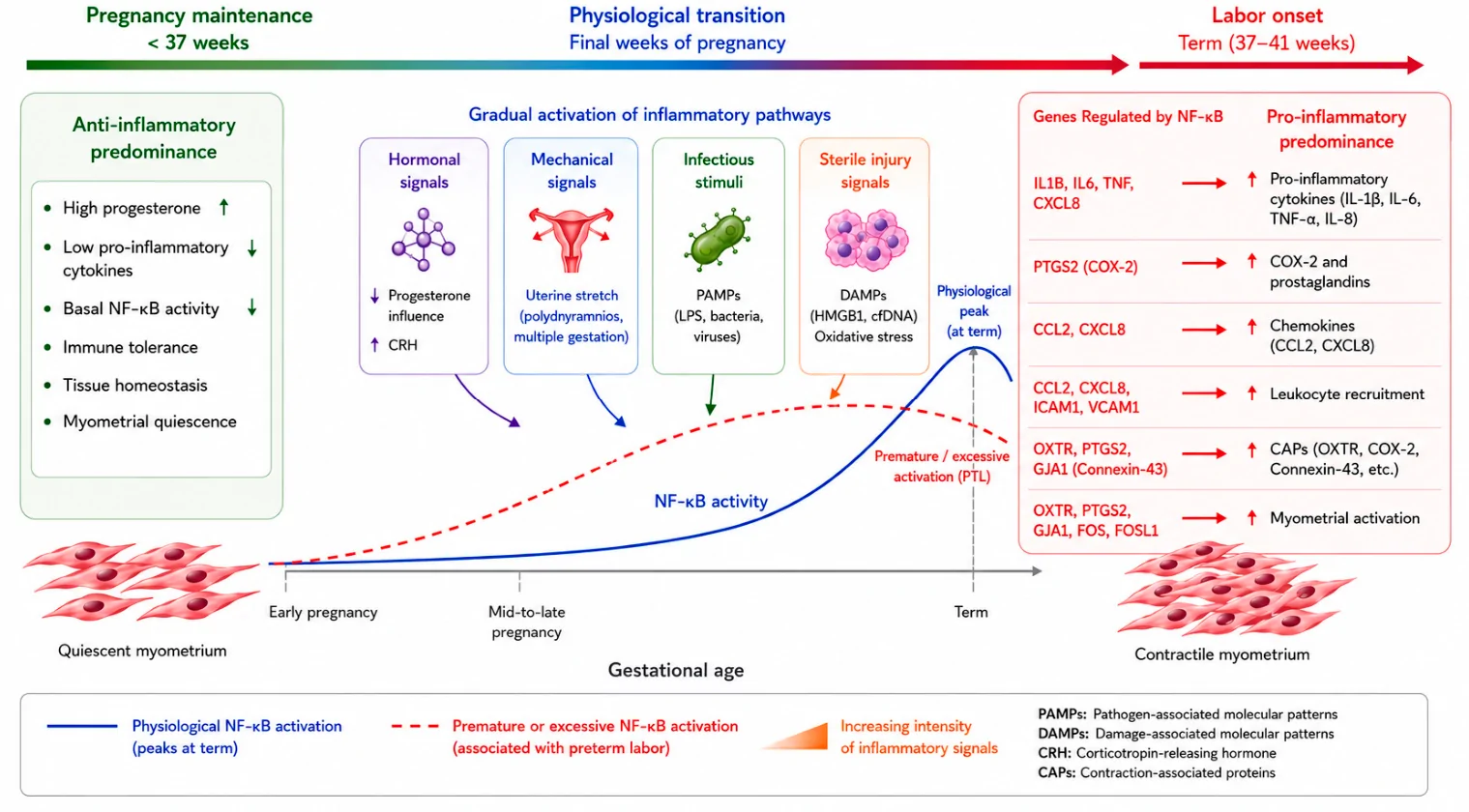
Transition from pregnancy maintenance to labor. Pregnancy is maintained by a progesterone-dominated anti-inflammatory environment with restrained NF-κB activity. Near term, physiological activation of NF-κB initiates labor, whereas premature or excessive activation of the same pathways promotes inflammation-associated preterm labor.

As term approaches (≥37 weeks of gestation), a coordinated and tightly regulated increase in pro-inflammatory signaling occurs. This includes activation of NF-κB-dependent pathways, which gradually overcomes pregnancy-maintaining mechanisms and initiates the transition of the uterus toward a contractile phenotype [4,5] (Figure 1).

This transition converts the myometrium from a quiescent to a contractile state, representing a key step in labor initiation. Concurrently, the immunological balance shifts toward a pro-inflammatory environment that facilitates uterine contractions, cervical ripening, and fetal membrane rupture, reflecting a physiological inflammatory program of parturition [6,7].

In the final stages of pregnancy, the expression of pro-inflammatory cytokines and chemokines increases, triggering a coordinated cascade of events required for labor onset. The NF-κB signaling pathway is central to integrating these inflammatory and stress-related signals, and to regulating the expression of genes responsible for initiating both physiological term labor and, when dysregulated, preterm labor [8].

### 2.2. Key Cytokines and Immune Cells in Labor Regulation

As term approaches, immune cells, including neutrophils, macrophages, NK cells, and T lymphocytes, become increasingly activated within gestational tissues. Through NF-κB-dependent signaling, these cells induce the expression of pro-inflammatory mediators (interleukin 1 beta [IL-1 β], interleukin 6 [IL-6], interleukin 8 [IL-8], tumor necrosis factor alfa [TNF-α], and cyclooxygenase 2 [COX-2]), which regulate contraction-associated proteins (gap junction alpha 1, oxytocin receptor [OXTR], and COX-2) and promote prostaglandin synthesis.

These changes enhance myometrial excitability and increase calcium influx into mSMCs, thereby promoting coordinated uterine contractility. COX-2 additionally contributes to the induction of matrix metalloproteinases (MMPs), particularly MMP-9, which remodel the extracellular matrix and weaken fetal membranes, supporting membrane rupture and progression of labor [4,9].

Elevated levels of cytokines and chemokines (C-X-C motif chemokine ligand 1 [CXCL1], C-X-C motif chemokine ligand 8 [CXCL8], and C-C motif chemokine ligand 2 [CCL2]) promote leukocyte recruitment—mainly macrophages and neutrophils—to the myometrium, cervix, and fetal membranes, establishing a positive feedback loop that sustains and intensifies the local inflammatory response during labor [6,8].

Corticotropin-releasing hormone (CRH), primarily produced by the placenta, plays a pivotal role in regulating gestational length. Elevated CRH levels are associated with placental dysfunction and fetal maturation, while maternal stress further activates the hypothalamic–pituitary–adrenal axis, increasing cortisol and CRH production. This, in turn, stimulates the release of pro-inflammatory cytokines, including IL-6, IL-1β, and TNF-α, contributing to activation of the inflammatory pathways involved in parturition [10] (Table 1).

### 2.3. Immunological Balance During Pregnancy

Normal pregnancy depends on the maintenance of immunological balance, with anti-inflammatory mechanisms predominating throughout most of gestation. Progesterone suppresses pro-inflammatory pathways and maintains myometrial quiescence until late gestation, when functional progesterone withdrawal gradually reduces its anti-inflammatory influence.

The resulting shift toward a pro-inflammatory state initiates the physiological program of labor, with NF-κB activation serving as a central regulatory node. Studies have demonstrated that the dimer composition of NF-κB activity varies depending on physiological status. In non-pregnant women, p50 homodimers predominate, whereas during pregnancy and especially during labor, p65:p50 heterodimers become more prevalent. This change reflects a shift from a transcriptional profile that is primarily anti-inflammatory to one that promotes the pro-inflammatory and contractile processes necessary for the initiation of labor during its physiological course [4,7,9].

### 2.4. Role of Immunological Disturbances in Labor and Organ Changes During Pregnancy

Premature disruption of immunological balance can shift the tightly regulated inflammatory milieu of pregnancy toward an enhanced pro-inflammatory state, increasing the intensity or advancing the timing of NF-κB activation along a physiological–pathological continuum and thereby promoting inappropriate initiation of the labor program [9]. Such dysregulation does not necessarily reflect a qualitatively distinct process but rather an amplification or temporal misalignment of pathways that normally participate in term parturition, ultimately increasing the risk of PTB [4].

Triggers include intrauterine infections, dysbiosis, oxidative stress, tissue ischemia, excessive NF-κB activation, and uterine overdistension in cases of polyhydramnios or multiple gestation [4,9]. In the chorioamniotic membranes, high mobility group box 1 regulates embryo implantation, fetal development, and the timing of labor onset. Its overexpression has been associated with miscarriage, PTB, and preeclampsia by inducing inflammation via the NF-κB pathway. This further amplifies inflammation through inflammasome activation (nucleotide-binding oligomerization domain-containing protein 1/2-like receptor family pyrin domain containing 3 [NLRP3], absent in melanoma 2 NOD, NOD1/2), caspase-1, MMP-9, and transcriptional upregulation of inflammatory genes.

Together, these pathways illustrate how both mechanical and molecular danger signals converge on NF-κB to modulate inflammatory activation in the amnion, depending on their magnitude and duration [11,12,13]. When this inflammatory cascade is prematurely initiated or excessively amplified, it can lead to labor, including leukocyte infiltration and increased expression of IL-1α, IL-1β, IL-6, and TNF-α, chemokines (CCL2 and CXCL8), and COX-2. This graded increase in inflammatory signaling enhances prostaglandin synthesis and calcium influx into the myometrium, which, if occurring earlier than developmentally appropriate, may precipitate premature uterine contractility and preterm labor [8,14].

During pregnancy, numerous organ changes occur, primarily in the thymus. The thymus undergoes involution (a decrease in total organ mass), the number of T lymphocytes decreases, and the stroma undergoes remodeling. This is due to progesterone, estrogen, steroids, and RANK/RANKL [15].

## 3. Description of the NF-κB Pathway

### 3.1. Structure and Activation of NF-κB

The NF-κB pathway is a key regulator of inflammation and is implicated in numerous diseases, including autoimmune disorders, respiratory diseases, cancer, and diabetes [16,17,18]. In pregnancy, it also plays a critical and tightly regulated role in coordinating the immune, mechanical, and endocrine signals required for parturition. Understanding its structure and activation mechanisms is therefore essential for elucidating both physiological labor and inflammation-driven preterm birth [19]. In gestational tissues, NF-κB signaling operates within a unique immunological context characterized by the need to balance pro-inflammatory activation with maternal–fetal tolerance, distinguishing it from classical inflammatory responses observed in non-pregnant tissues.

Nuclear factor kappa B (NF-κB) comprises a family of five transcription factors: NF-κB1 (p105/p50), NF-κB2 (p100/p52), p65 (RelA), Rel homolog B (RelB), and c-Rel [20,21]. NF-κB regulates inflammatory responses, immune function, cell survival, and proliferation through the expression of genes such as cyclin A, cyclin D1, and cyclin-dependent kinase 6 (CDK6). Activation of NF-κB also protects cells from TNF-α-induced apoptosis, supporting tissue integrity and cell survival; however, when dysregulated, it may contribute to chronic inflammation and oncogenesis.

The pathway is most commonly activated via the classic p65/p50 heterodimer. In its inactive state, NF-κB is retained in the cytoplasm by the nuclear factor kappa B inhibitor (IκB). It exists in three forms (IκB-α, IκB-β, and IκB-ϵ). Its role is to mask the nuclear localization signal (NLS) and prevent gene transcription.

Upon stimulation by cytokines (TNF-α and IL-1β), DNA damage, oxidative stress, Toll-like receptor (TLR) activation (via lipopolysaccharide (LPS), pathogen-associated molecular patterns [PAMPs], or damage-associated molecular patterns [DAMPs]), or antigen receptor signals (T-cell receptor [TCR]/B-cell receptor), this inhibitory complex is disrupted [17,20,21,22].

Activation begins with stimulation of the IκB kinase (IKK) complex, composed of two catalytic subunits (IKKα and IKKβ) and a regulatory subunit (IKKγ/NEMO). IKK phosphorylates IκB, leading to its ubiquitination and proteasomal degradation. This exposes the NLS, allowing NF-κB to translocate to the nucleus, where it binds specific DNA sequences to initiate transcription of genes encoding pro-inflammatory cytokines (IL-6, IL-8, and TNF-α), COX-2, immunoregulatory molecules (intercellular adhesion molecule 1, major histocompatibility complex, OXTR, and cell-survival proteins (B-cell lymphoma 2 and Bcl-xL)) [20,21,22].

In the context of pregnancy, the downstream transcriptional targets of NF-κB extend beyond classical inflammatory mediators to include genes directly involved in uterine activation, highlighting its dual role as both an immune regulator and a key driver of parturition-specific processes. A single pro-inflammatory stimulus, such as IL-1β, can trigger additional cytokine production (e.g., IL-6) and upregulate COX-2 expression, forming a positive inflammatory amplification loop. At the same time, NF-κB induces transcription of IκB, which re-sequesters NF-κB in the cytoplasm, providing an intrinsic negative feedback mechanism that limits excessive pathway activation [16,17,18,19,20,21]. Despite extensive characterization of NF-κB signaling, the precise mechanisms that determine the transition from physiological to pathological activation in pregnancy remain incompletely understood, representing an important area for future research.

### 3.2. Activation Pathways

Two main NF-κB activation pathways are recognized:

(1) Canonical pathway: Activated by TNF-α, IL-1β, LPS, oxidative stress, PAMPs, and DAMPs via receptors such as TNFR, IL-1R, and TLRs (mainly TLR4). TLR expression is highest in monocytes, macrophages, and neutrophils, which serve as major cytokine sources during early inflammatory responses. This pathway involves phosphorylation of the IKK complex, predominantly IKKβ and IKKγ, and primarily employs the p65/p50 heterodimer, producing a rapid and transient inflammatory response [17,18,19,20,21,23,24]. In gestational tissues, activation of the canonical pathway is most commonly associated with acute inflammatory stimuli, including infection and sterile inflammatory signals, and is therefore considered a principal driver of inflammation-induced preterm labor.

(2) Non-canonical pathway: Activated by B-cell Activating Factor (BAFF), CD40L, and lymphotoxin-β (LT-β) via the B-cell Activating Factor Receptor (BAFF-R), CD40, and LTβR receptors. These signals prevent the ubiquitin-mediated degradation of NF-κB-inducing kinase (NIK), which consequently leads to the activation of IKKα independently of IKKγ. NIK phosphorylates the p100 protein, causing its partial degradation to the p52 protein. The p52 protein binds to RelB to form the RelB/p52 dimer, which is predominant in this pathway and induces a slower and more sustained transcriptional response compared to the canonical pathway [17,18,19,20,21,24].

### 3.3. NF-κB Activation at Term and in Preterm Labor

Studies have demonstrated a marked increase in NF-κB activity in the uterine decidua and placenta preceding and during labor, highlighting its fundamental role in parturition. NF-κB acts as a central integrator of multiple signaling pathways that coordinate the transition of the myometrium from a quiescent to a contractile state. In this context, NF-κB can be viewed not only as an inflammatory mediator but also as a key regulatory hub that integrates endocrine, mechanical, and immune signals to determine the timing of labor onset.

In the uterus, NF-κB activation is driven by several overlapping and partly physiological mechanisms:

(1) TLR4 activation: Classically associated with infection via LPS, but also detected in preterm labor without overt infection, likely reflecting responses to sterile inflammatory stimuli such as mechanical stretch, hypoxia, or ischemic injury, which promote the release of endogenous DAMPs.

(2) Pro-inflammatory cytokines: IL-1β and TNF-α, which are involved in both physiological labor at term and inflammation-associated preterm labor.

(3) Mechanical signals: Uterine stretching activates NF-κB and Mitogen-Activated Protein Kinases pathways, contributing to the normal preparation of the uterus for labor.

The convergence of these distinct upstream signals on a common NF-κB-dependent transcriptional program underscores the role of this pathway as a central integrator of both physiological and pathological triggers of labor. These converging signals induce the expression of contraction-associated proteins (CAPs), including:

- Increased OXTR expression, enhancing myometrial sensitivity to oxytocin;
- Enhanced COX-2 expression, leading to increased prostaglandin synthesis (PGF_2_α via FP receptors and PGE_2_ via prostaglandin E receptor 1/3 [EP1/EP3]);
- Upregulation of connexin-43, facilitating electrical coupling and synchronized myometrial contractions;
- Chemokine production (CCL2 and CXCL8/IL-8), promoting leukocyte recruitment and local immune activation.

These processes support the physiological onset and progression of labor. NF-κB activation is observed in all pregnancies prior to labor onset and is considered a normal component of the parturition program. The distinction between term and preterm labor appears to depend not on the presence or absence of NF-κB activation, but rather on its timing, magnitude, and regulatory context.

Under certain conditions—such as intrauterine infection or exaggerated inflammatory signaling—this tightly regulated pathway may become activated earlier or more robustly, contributing to the premature initiation of uterine contractions and the inflammation characteristic of preterm labor [18,23,25,26].

Although most studies focus on the canonical NF-κB pathway, there is considerable evidence suggesting that the non-canonical pathway also plays an important role in preterm birth, and that the two pathways are closely interrelated. In this model, the canonical NF-κB pathway initiates inflammation, while the non-canonical pathway prevents its resolution. The canonical activation pathway acts rapidly but cannot be sustained for too long, as it would lead to tissue damage. The body activates a regulatory mechanism in the form of the non-canonical NF-κB pathway. The failure of the stimulus to subside (sterile inflammation, persistent infection) causes cells to secrete ligands for the TNF receptor family (CD40L, LIGHT, and BAFF), which activates the non-canonical pathway. The non-canonical pathway is responsible for the chronic production of chemokines and, consequently, for leukocyte migration. Influxing macrophages contribute to cervical remodeling via metalloproteinases and collagen degradation. In addition, migrating leukocytes secrete cytokines that enhance the canonical NF-κB pathway [27,28].

## 4. NF-κB Pathway Modulators as Potential Tocolytic Agents

### 4.1. Natural Inhibitors

#### 4.1.1. Curcumin

Curcumin, the main polyphenol in *Curcuma longa*, is a natural modulator of NF-κB signaling with well-documented anti-inflammatory properties relevant to inflammation-associated preterm birth. In vitro and ex vivo studies demonstrate that curcumin attenuates NF-κB activation in gestational tissues by inhibiting IKKα/β, stabilizing IκB, and reducing p50/p65 nuclear translocation, leading to decreased IL-6 expression and gp130/Signal Transducer and Activator of Transcription 3 (STAT3) signaling [29]. Similar effects have been observed in placental, fetal membrane, and myometrial explants, including reduced p65 DNA binding, oxidative stress, COX-2 expression, cytokine production, and prostaglandin release [30].

In preclinical models of LPS-induced PTB, curcumin reduced NF-κB activation, cytokine levels, and the incidence of preterm birth, supporting its role in modulating excessive rather than basal inflammatory signaling [31]. Additional studies indicate systemic anti-inflammatory effects and inhibition of TLR4/NF-κB signaling in vivo [32]. Curcumin has also been shown to reduce myometrial contractility in vitro, suggesting potential tocolytic properties [33]. Systematic reviews have identified curcumin as a potential candidate for further investigation, particularly in the context of TLR4–NF-κB modulation [34].

#### 4.1.2. Resveratrol

Resveratrol is a natural polyphenol that modulates NF-κB signaling and reduces pro-inflammatory cytokine production in trophoblast cells [35]. In preclinical PTB models, it decreases iNOS and COX-2 expression, limits prostaglandin synthesis, and reduces preterm birth incidence [36,37]. Additionally, ex vivo studies demonstrate direct myometrial relaxant effects, indicating potential dual anti-inflammatory and tocolytic activity [38].

#### 4.1.3. Omega-3 Fatty Acids

Omega-3 fatty acids (docosahexaenoic acid, eicosapentaenoic acid) exert anti-inflammatory effects partly through NF-κB modulation. In gestational tissues, they reduce cytokine production and attenuate infection-induced inflammation by decreasing IκB phosphorylation and IKK activity [39,40]. Clinical evidence remains limited, although some studies suggest reduced placental inflammation with supplementation [41]. Reviews further indicate inhibition of TLR–NF-κB signaling and downstream mediators such as COX-2 and prostaglandins [42]. Specialized pro-resolving mediators derived from omega-3 fatty acids contribute to inflammation resolution; for example, resolving E3 reduces NF-κB activation and PTB incidence in experimental models [43,44].

#### 4.1.4. Other Natural Compounds

Additional natural compounds—including apigenin, quercetin, epigallocatechin gallate (EGCG), ginger-derived compounds, and luteolin—demonstrate NF-κB inhibitory activity and reduction in pro-inflammatory mediators in gestational tissues [30,45,46,47,48]. Some also exhibit direct effects on myometrial contractility. Natural compounds appear to modulate excessive NF-κB activation rather than fully suppress the pathway, although the evidence remains largely preclinical and requires further validation.

**Figure 2:**
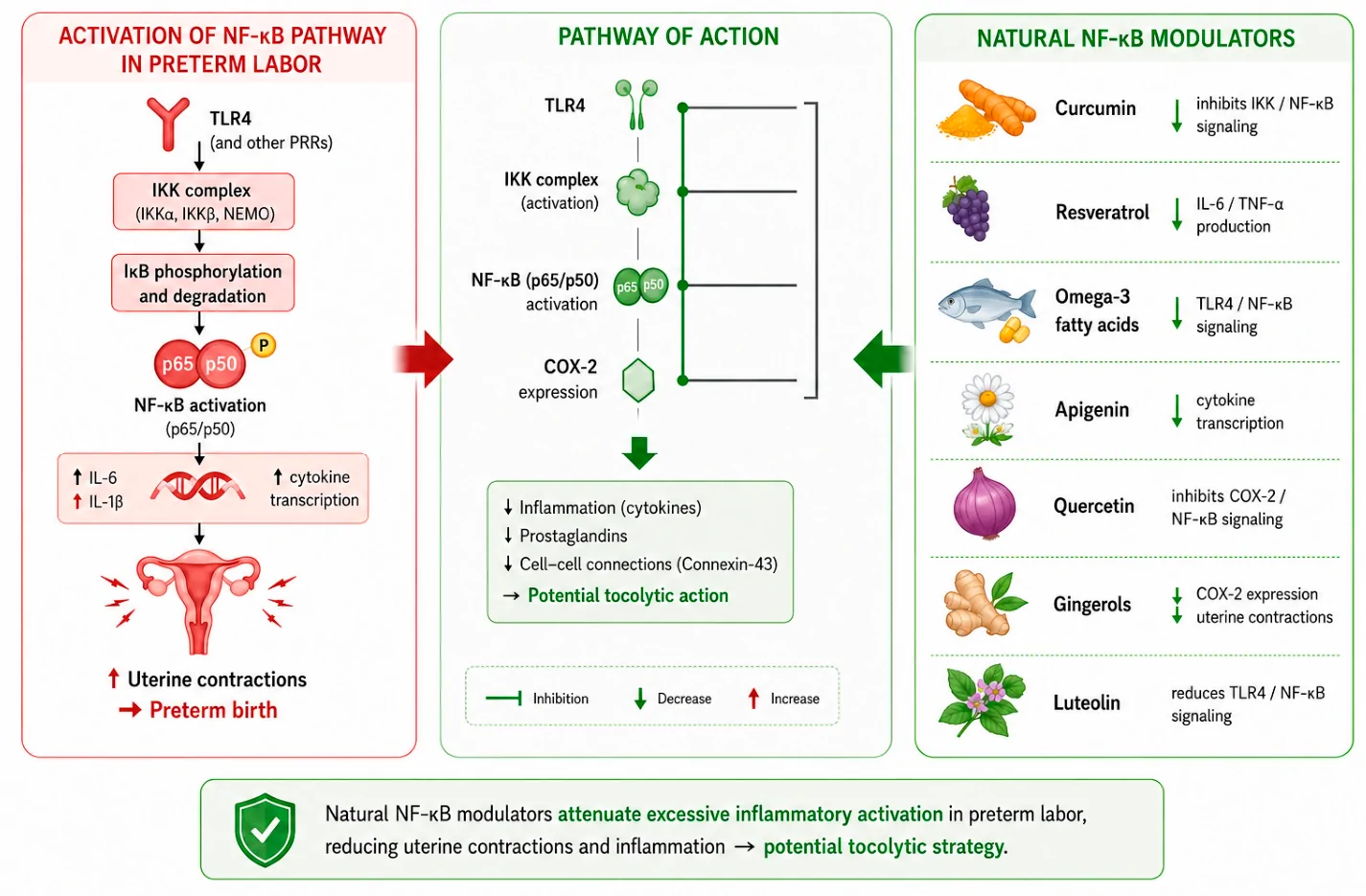
Schematic overview of NF-κB pathway activation in preterm labor and its modulation by natural compounds.

Figure 2 illustrates the potential mechanisms by which natural NF-κB modulators interfere with inflammatory activation in preterm labor.

#### 4.1.5. Pregnancy-Specific Pharmacokinetics and Bioavailability of Drugs

Although several natural compounds, including curcumin and resveratrol, have shown promising anti-inflammatory effects in experimental models, their clinical application is limited by low oral bioavailability and rapid metabolism. In addition, pregnancy-specific pharmacokinetics, placental transfer, fetal exposure, and long-term fetal safety remain insufficiently characterized. Further studies are needed to determine the optimal dosing strategies and therapeutic windows before these agents can be translated into clinical practice [33,35,42,49].

### 4.2. Synthetic Inhibitors

#### 4.2.1. IKK Kinase Inhibitors

Selective IKKβ inhibitors, such as SC-514 and TPCA-1, attenuate NF-κB activation and reduce pro-inflammatory mediators in gestational tissues and animal models, supporting their role in limiting inflammation-driven uterine activation [50,51]. Reviews suggest that these agents may offer a more targeted approach compared to nonspecific anti-inflammatory therapies [52,53].

#### 4.2.2. Sulfasalazine

Sulfasalazine reduces NF-κB activation and cytokine expression in gestational tissues [51]; however, paradoxical pro-inflammatory effects and increased apoptosis have also been reported [54]. Clinical observations do not indicate an increased PTB risk, but preclinical data remain inconsistent, limiting its therapeutic potential [55].

#### 4.2.3. TLR4 Inhibitors

TLR4 antagonists, including (+)-naloxone and (+)-naltrexone, reduce cytokine production and prevent inflammation-induced preterm labor in animal models, highlighting the potential of targeting upstream NF-κB activation [56,57]. Reviews support TLR4 as a relevant therapeutic target in inflammation-associated PTB [58,59].

#### 4.2.4. Modern Synthetic Modulators

Recent approaches focus on more selective modulation of inflammatory signaling. The Bromodomain and Extra-Terminal proteins (BET) inhibitor JQ1 reduces inflammatory gene expression and delays labor onset [60]. The NLRP3 inhibitor Matthew Cooper Compound 950 (MCC950) suppresses inflammasome activation, reduces inflammation, and prevents PTB in experimental models [61,62].

Additional compounds, including aprotic amides, inhibit NF-κB activation and cytokine expression [63], while exosome-based delivery systems may improve tissue-specific targeting and preserve physiological NF-κB functions [64].

Table 2 summarizes the classification of NF-κB modulators according to the mechanism of action and their effects in preterm birth (Table 2).

Overall, natural and synthetic NF-κB modulators differ primarily in pharmacological profile rather than ultimate biological target. Natural compounds generally exert multi-target, pleiotropic anti-inflammatory effects with relatively modest potency, which may confer safety advantages but limits precise pathway control. In contrast, synthetic inhibitors provide more selective and robust targeting of specific components of the NF-κB signaling cascade, enabling stronger suppression of inflammation-associated pathways implicated in preterm labor, albeit with a potentially narrower therapeutic window. Taken together, these differences underscore that future translational strategies should prioritize balanced, context-dependent modulation of NF-κB activity rather than complete pathway inhibition.

Table 3 summarizes a mechanistic classification of NF-κB modulators according to their primary molecular targets within the signaling pathway, complementing the functional classification presented in Table 2 [65] (Table 3).

### 4.3. Limitations on the Use of NF-kappaB Inhibitors

Given the wide range of functions of the NF-kappaB pathway in regulating the immune system, systemic inhibition may lead to immunosuppression. This pathway is, in fact, an essential regulator of the activation and survival of innate immune cells (such as neutrophils and macrophages) as well as T- and B-cell-mediated immune responses. Consequently, nonselective inhibition of the NF-kappaB pathway may increase the risk of bacterial, viral, and fungal infections. This is particularly important in pregnant women, in whom physiological adaptation of the immune system occurs to enable maternal–fetal tolerance. Equally important are the long-term consequences for the mother in the form of impaired immune surveillance against neoplastic transformation [66,67]. It is also important to note that NF-κB pathway activity plays a significant role in the initiation of physiological labor, and its inhibition may disrupt this process [68]. These concerns highlight the need to develop tissue-specific therapeutic strategies, which could address the aforementioned issues and serve as an effective means of preventing PTB.

## 5. Materials and Methods

This study was conducted as a narrative review of the literature to summarize current evidence regarding the role of inflammatory processes and nuclear factor kappa B (NF-κB) signaling in term and preterm labor, with particular emphasis on the therapeutic potential of NF-κB modulation. A comprehensive literature search was performed to identify relevant publications; however, the review was not designed as a systematic review and did not follow a formal PRISMA protocol. Owing to the heterogeneity of the included studies with respect to experimental models, study designs, and reported outcomes, the evidence was synthesized narratively rather than quantitatively.

The literature search was performed using the PubMed, Scopus, Web of Science, and Embase databases. The primary search included articles published between January 2015 and June 2026. Earlier landmark publications were additionally included where necessary to provide historical context and mechanistic background. The final database search was conducted in June 2026.

The literature search combined Medical Subject Headings (MeSH) and free-text keywords related to NF-κB signaling, parturition, preterm birth, inflammation, cytokines, prostaglandins, and gestational tissues, including the myometrium, placenta, fetal membranes, amnion, and decidua. Equivalent keyword combinations were adapted for each database according to its indexing system. Boolean operators (AND and OR) were used to combine search terms where appropriate. Searches were limited to English-language articles published between January 2015 and June 2026. Duplicate references were excluded where appropriate during the literature selection process.

Eligible publications comprised original in vitro, ex vivo, animal, translational, and clinical studies, as well as relevant review articles published in peer-reviewed English-language journals. Studies were included if they investigated NF-κB signaling in the context of labor, inflammatory mechanisms involved in parturition, or pharmacological modulation of these pathways. Review articles were primarily used to identify additional relevant primary studies and to provide contextual background. The analysis was based on data derived exclusively from original research articles. If a primary study appeared both as an original publication and within a review article, only the original publication was included in the analysis. Conference abstracts, duplicate publications, non-English articles, and studies not directly relevant to the review topic were excluded.

Relevant publications were selected by the authors based on their relevance to the objectives of the review.

Given the methodological heterogeneity of the included publications, a formal assessment of study quality and risk of bias was not performed. This was a deliberate methodological decision made during the design of the review, as the included evidence comprised different types of studies, including in vitro, ex vivo, animal, translational, clinical, and review articles. Because no single validated tool can be applied consistently across such diverse study designs, the findings were synthesized using a qualitative narrative approach. Throughout the review, the available evidence was interpreted with consideration of the limitations of the individual studies.

## 6. Conclusions and Future Perspectives

### 6.1. Key Conclusions

Inflammation is a fundamental driver of both physiological and pathological labor, with NF-κB acting as a central molecular hub that integrates hormonal, mechanical, infectious, and sterile inflammatory signals across gestational tissues. Appropriate activation of NF-κB is essential for normal term parturition, whereas its premature or excessive activation contributes to inflammation-driven preterm labor through the induction of cytokines, prostaglandins, chemokines, and contraction-associated proteins.

Current evidence identifies NF-κB as one of the most promising mechanistic targets for limiting pathological uterine inflammation while preserving the physiological signaling required for timely labor.

### 6.2. Limitations of Current Evidence

Despite substantial progress in understanding NF-κB biology during pregnancy, several important limitations remain. Most available evidence is derived from in vitro studies and animal models, whereas well-designed clinical studies evaluating NF-κB-targeted interventions during pregnancy are scarce. Furthermore, considerable heterogeneity exists among experimental models, inflammatory stimuli, gestational tissues, dosing regimens, and outcome measures, making direct comparison between studies difficult.

In addition, because NF-κB is a pleiotropic transcription factor involved in immune homeostasis, fetal development, and normal parturition, complete inhibition of this pathway is unlikely to be clinically desirable. Consequently, the translational relevance of many experimental findings remains uncertain.

### 6.3. Future Perspectives

Future research should move beyond demonstrating anti-inflammatory activity toward identifying tissue-specific, cell-specific, and context-dependent mechanisms of NF-κB regulation during pregnancy. Particular emphasis should be placed on defining therapeutic windows, developing targeted drug-delivery systems that minimize fetal exposure, and integrating transcriptomic, proteomic, and single-cell approaches to characterize NF-κB signaling within individual gestational compartments. We must also ask whether tissue-specific inhibition of the NF-κB pathway is possible. If so, this should be the subject of future research.

Equally important is the need for well-designed translational studies and randomized clinical trials to establish the pharmacokinetics, safety, and efficacy of NF-κB modulators in pregnant women.

### 6.4. Final Perspective

Rather than global suppression of NF-κB signaling, selective modulation of excessive pathological activation appears to represent the most biologically plausible therapeutic strategy. Natural compounds and emerging synthetic modulators provide encouraging preclinical evidence that excessive inflammation can be attenuated without abolishing the physiological functions of NF-κB required for normal pregnancy and term labor.

Although these findings support NF-κB as a compelling therapeutic target, clinical translation remains at an early stage. Future advances will depend on the development of selective, pregnancy-compatible interventions capable of restoring inflammatory homeostasis while maintaining maternal and fetal safety.

## Figures and Tables

**Table 1:** Key cytokines, chemokines, and contraction-associated proteins (CAPs) in the regulation of labor.

Category	Key Molecules	Role in Labor
Proinflammatory Cytokines	IL-1β, IL-6, TNF-α	Promote myometrial contractions; induce COX-2 expression and prostaglandin synthesis
Chemokines	CXCL1, CXCL8, CCL2	Mediate leukocyte recruitment to the myometrium and fetal membranes, amplifying the inflammatory response
Contraction-Associated Proteins (CAPs)	OXTR, Connexin-43, COX-2	Enhance myometrial contractility and synchronize uterine contractions
Extracellular Matrix (ECM) Enzymes	MMP-9	Remodel the extracellular matrix and weaken fetal membranes, facilitating labor onset

**Table 2:** Classification of NF-κB modulators according to mechanism of action and effects in preterm birth (PTB).

Category	Natural Modulators	Synthetic Modulators
Examples	Curcumin, Resveratrol, Omega-3 fatty acids, Apigenin, Quercetin, EGCG	SC-514, TPCA-1, Sulfasalazine, JQ1, MCC950
Mechanism of action	Inhibition of NF-κB activation via suppression of IKK activity and TLR4/NF-κB signaling; reduction in pro-inflammatory cytokine production	Selective inhibition of IKKβ, NLRP3 inflammasome, and BET proteins; suppression of p65 phosphorylation and NF-κB transcriptional activity
Effects in PTB	Decreased expression of IL-1β, IL-6, TNF-α, and COX-2; attenuation of myometrial contractility	Reduced prostaglandin and cytokine production; delay or prevention of inflammation-induced preterm labor

**Table 3:** Classification of modulators according to their primary targets within the NF-κB signaling pathway.

Class of NF-κB Modulators	Mechanism of Action	Representative Compounds
IKK inhibitors	Inhibit IKKα/IKKβ-mediated phosphorylation of IκB, preventing its degradation and downstream NF-κB activation	TPCA-1, SC-514, BMS-345541, IMD-0354, Sulfasalazine, BAY 11-7082
IκB stabilizers	Prevent proteasomal degradation of IκB, thereby retaining NF-κB in the cytoplasm	MG-132, Bortezomib, NBD peptide, and aprotic amides (DEA, DPA, DMA)
NF-κB nuclear translocation inhibitors	Block nuclear import of p65/p50 complex or interfere with DNA binding to promoters	SN50 peptide, Parthenolide, CAPE, Curcumin, Resveratrol, MCC950
TLR4/inflammasome-related NF-κB modulators	Reduce upstream TLR4-mediated activation of NF-κB signaling	(+)-Naltrexone and(+)-Naloxone

## Data Availability

No new data were created or analyzed in this study. Data sharing is not applicable to this article.

## Abbreviations

The following abbreviations are used in this manuscript:

BAFF	B-cell Activating Factor
BAFF-R	B-cell Activating Factor Receptor
BET	Bromodomain and Extra-Terminal proteins
BRD	Bromodomain-containing protein
CAPs	Contraction-associated proteins
CCL2	C-C motif chemokine ligand 2
CD40L	CD40 ligand
CDK6	Cyclin-dependent kinase 6
COX-2	Cyclooxygenase-2
CRH	Corticotropin-releasing hormone
CXCL1	C-X-C motif chemokine ligand 1
CXCL8 (IL-8)	C-X-C motif chemokine ligand 8
DAMPs	Damage-associated molecular patterns
EGCG	Epigallocatechin gallate
EP	Prostaglandin E receptor
FP	Prostaglandin F receptor
gp130	Glycoprotein 130
IKK	IκB kinase
IKKα	IκB kinase alpha
IKKβ	IκB kinase beta
IKKγ (NEMO)	IκB kinase gamma/NF-κB Essential Modulator
IL-1α	Interleukin 1 alpha
IL-1β	Interleukin 1 beta
IL-6	Interleukin 6
IL-8	Interleukin 8
iNOS	Inducible Nitric Oxide Synthase
JQ1	BET bromodomain inhibitor JQ1
LTβ	Lymphotoxin beta
LTβR	Lymphotoxin beta receptor
LPS	Lipopolysaccharide
MCC950	Matthew Cooper Compound 950
MMP-9	Matrix metalloproteinase-9
mSMC	Myometrial smooth muscle cells
NF-κB	Nuclear factor kappa B
NIK	NF-κB-inducing kinase
NK cells	Natural killer cells
NLRP3	NOD-like receptor family pyrin domain containing 3
NLS	Nuclear localization signal
NOD1/2	Nucleotide-binding oligomerization domain-containing protein ½
OXTR	Oxytocin receptor
PAMPs	Pathogen-associated molecular patterns
PGF_2_α	Prostaglandin F2 alpha
PGE_2_	Prostaglandin E2
PTB	Preterm birth
RCT	Randomized Controlled Trial
RelA (p65)	v-rel reticuloendotheliosis viral oncogene homolog A
RelB	v-rel reticuloendotheliosis viral oncogene homolog B
ROS	Reactive Oxygen Species
TLR4	Toll-like receptor 4
TNF-α	Tumor necrosis factor alpha
TCR	T-cell receptor
